# Long-Term Humoral Response After a Second Dose of SARS-CoV-2 mRNA Vaccine in Japanese Kidney Transplant Recipients

**DOI:** 10.3389/fmicb.2022.922042

**Published:** 2022-06-09

**Authors:** Yutaro Ohki, Mayuko Kawabe, Izumi Yamamoto, Haruki Katsumata, Yasuyuki Nakada, Akimitsu Kobayashi, Fumihiko Urabe, Jun Miki, Hiroki Yamada, Takahiro Kimura, Nanae Matsuo, Yudo Tanno, Tetsuya Horino, Ichiro Ohkido, Hiroyasu Yamamoto, Takashi Yokoo

**Affiliations:** ^1^Division of Nephrology and Hypertension, Department of Internal Medicine, The Jikei University School of Medicine, Tokyo, Japan; ^2^Department of Urology, The Jikei University School of Medicine, Tokyo, Japan; ^3^Department of Infectious Disease and Infection Control, The Jikei University School of Medicine, Tokyo, Japan

**Keywords:** SARS-CoV-2 vaccine, COVID-19, kidney transplant recipients, humoral response, anti-S SARS-CoV-2 IgG antibody titers

## Abstract

**Background:**

The mortality rate due to COVID-19 in kidney transplant recipients (KTRs) is 16.8 to 32%. Vaccination against COVID-19 is expected to contribute to the prevention of infection, severe disease, and mortality; however, it has been reported that the humoral response to the severe acute respiratory syndrome coronavirus 2 (SARS-CoV-2) mRNA vaccine in KTRs is poor. Vaccination strategies against COVID-19 vary from country to country, and in Japan, the third dose is given 6 months after the second dose. Few studies have evaluated long-term humoral responses after the second dose of SARS-CoV-2 mRNA vaccine. In addition, the superiority of BNT162b2 vaccine and mRNA-1,273 vaccine in KTRs regarding humoral response is controversial.

**Methods:**

Ninety-four KTRs were administered a second dose of the BNT162b2 or mRNA-1,273 vaccines, and anti-spike (anti-S) and anti-nucleocapsid (anti-N) SARS-CoV-2 antibody levels were measured 5 months (149.2 ± 45.5 days) later. The cutoff value of anti-S antibodies was defined ≥50 AU/ml and 1.4 Index for anti-N antibodies. The primary outcome was the rate of seropositivity, and factors associated with an appropriate humoral response were assessed by univariate and multivariate analysis.

**Results:**

Of 94 KTRs, only 45 (47.9%) patients were positive for anti-S antibodies. The median anti-S SARS-CoV-2 IgG antibody titers was 35.3 (Interquartile range 3.8 to 159.7). Anti-N SARS-CoV-2 IgG antibodies in all patients were < 1.4 Index. Response to SARS-CoV-2 mRNA vaccines were 43.2 and 65% for BNT162b2 and mRNA-1,273, respectively (*p* = 0.152). In comparison with high-dose, low-dose of mycophenolic acid was a robust factor associated with an adequate humoral response.

**Conclusion:**

The long-term humoral response after a second dose of SARS-CoV-2 mRNA vaccine in Japanese KTRs was poor. In comparison with high-dose, low-dose mycophenolic acid was related to an appropriate humoral response. Five months is too long to wait for a 3rd dose after 2nd dose of SARS-CoV-2 vaccine in KTRs. In this cohort, there was no statistical difference in humoral response to the BNT162b2 and mRNA-1,273 vaccines. Additional large observational studies and meta-analyses are needed to clarify the factors related to an appropriate humoral immune response to COVID-19 vaccination.

## Introduction

COVID-19, first reported in Wuhan, China, in December 2019, is an infectious disease caused by severe acute respiratory syndrome coronavirus 2(SARS-CoV-2) (Wu and McGoogan, 2020). The alpha and gamma strains of COVID-19 were the first variants of concern (VoCs) and have been replaced by the omicron strain. In Japan, BNT162b2 and mRNA-1,273 vaccines were approved on 14 February 14 2021 and 21 May, 2021, respectively, and vaccination has been vigorously promoted. Unfortunately, although kidney transplant recipients (KTRs) are vulnerable to COVID-19, the rate of appropriate response to a first dose of these vaccines in KTRs is 17% (Boyarsky et al., 2021a), and after the second dose is 46% (Benotmane et al., 2021b; Boyarsky et al., 2021b; Cucchiari et al., 2021; Grupper et al., 2021; Rozen-Zvi et al., 2021; Russo et al., 2022). Therefore, some countries have started a third vaccination for KTRs, the effectiveness of which has been demonstrated (Benotmane et al., 2021a; Bertrand et al., 2021; Hall et al., 2021; Kamar et al., 2021). Vaccination strategies against COVID-19 vary from country to country, and in Japan, the third dose is given 6 months after the second dose.

Few studies have evaluated long-term (average 5 months) humoral responses after the second dose of SARS-CoV-2 mRNA vaccine. Therefore, we analyzed the long-term humoral response after a second dose of SARS-CoV-2 mRNA vaccine in Japanese KTRs and identified the associated factors.

## Materials and Methods

### Patients

Ninety-four KTRs attending the Division of Nephrology and Hypertension at Jikei University Hospital (Tokyo, Japan) were enrolled in this prospective observational study. The BNT162b2 or mRNA-1,273 vaccine was administered twice to all patients. The vaccine type was not assigned in our hospital. Blood samples were collected during the visit, and the anti-spike (anti-S) SARS-CoV-2 IgG (cutoff value ≥50.0 AU/ml) and anti-nucleocapsid (anti-N) SARS-CoV-2 IgG (cutoff value 1.40 Index) levels were measured using SARS-CoV-2 IgG II Quant Kits (Abbott^©^) 5 months (149.2 ± 45.5 days) after the second dose. Previous studies have established the usefulness of the assay used here: cutoff values of ≥50 AU/ml for anti-S SARS-CoV-2 IgG and 1.4 for anti-N SARS-CoV-2 IgG, respectively, have been shown to clearly stratify SARS-CoV-2 recent infected patients and vaccinated patient (Narasimhan et al., 2021). Age, sex, body mass index (BMI), primary kidney disease leading to end-stage kidney disease, and past medical history were obtained from the medical records. Medication information, including antihypertensive drugs, immunosuppressants, and iron or 1-α-OH-D3 supplementation, was obtained from prescription records. Routine biochemical measurements included serum albumin, creatinine, as well as complete blood counts. The serum samples were stored at −80°C prior to use. The Ethics Committee of the School of Medicine of Jikei University [approval number 33–314(10934)] approved the study protocol. Written informed consent was obtained from all patients prior to inclusion in the study. Study procedures were performed in accordance with the Declaration of Helsinki and its revisions.

### Statistical Analysis

Based on the anti-S SARS-CoV-2 IgG antibody cutoff values, the cohort was divided into antibody-positive (≥ 50 AU/ml) and -negative groups (< 50 AU/ml). Continuous variables with normal and non-normal distributions were compared by unpaired *t*-tests and Mann–Whitney tests, respectively. Dichotomous variables were compared between the groups by chi-squared tests. Data are presented as means and standard deviations (SDs) for continuous variables with normal distributions or medians and interquartile ranges (IQR) for continuous variables with non-normal distributions. The dosage of mycophenolate mofetil (MMF) varies from patient to patient because the dosage is adjusted according to the development of infection after transplantation. TAC, on the other hand, is treated as a dichotomous variable because its dosage is determined by measuring blood TAC target levels. For methylprednisolone, the dose is constant at 5 mg every other day for all patients 1 year after transplantation in our institution. Therefore, only MMF is treated as a continuous variable. Factors associated with an appropriate humoral response were evaluated using univariate and multivariate logistic regression models. To confirm the same trend with a different statistical model, univariate and multivariate liner regression models were performed to identify factors independently associated with anti-S SARS-CoV-2 IgG titers. Anti-S SARS-CoV-2 IgG antibody titers were pre-processed by converting to common logarithms (log10) to adjust for normality of distribution. The risks were evaluated by odd ratios (OR) and coefficients, such as *β* with 95% confidence intervals (95% CI). Differences with a two-sided value of *p* < 0.05 were considered significant. Data were analyzed using Stata 17.0 (Stata Corp LP, College Station, TX).

## Results

**Figure 1:**
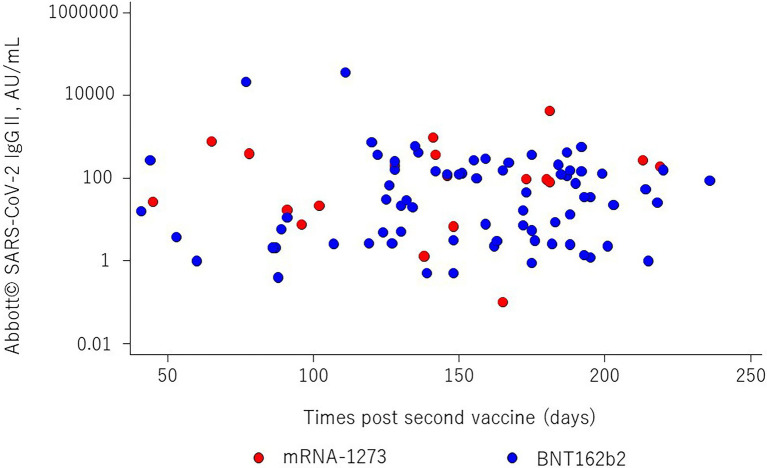
The relationship between SARS-CoV-2 IgG II antibody titers and times post second vaccine (days). The red and blue dots show mRNA-1,273 and BNT162b2, respectively. There is no statistical difference between two vaccines (*p* = 0.09).

The mean age was 53.7 ± 11.7 years, males comprised 67% of the study population, and the mean time elapsed since kidney transplantation was 10.7 (IQR = 4.4 to 16.9) years. The frequencies of BNT162b2 and mRNA-1,273 vaccination were 79.6 and 20.4%, respectively. The anti-S SARS-CoV-2 IgG antibody titers are shown in Figure 1. The median titer was 27.5 (IQR = 3.0 to 153.0) in BNT162b2 and 97.1 (IQR = 17.0 to 369.2) in mRNA-1,273, respectively. It was no significant differences in anti-S SARS-CoV-2 IgG titers by vaccine types (*p* = 0.09). Overall, the median SARS-CoV-2 IgG S antibody titer was 35.3 (IQR = 3.8 to 159.7), and the appropriate humoral response rate was 47.8% using a cutoff value of ≥50.0 AU/ml. The subjects were divided into two groups according to immunogenicity, and the clinical characteristics of the two groups are shown in Table 1. There were significant differences between the two groups in time since last transplant, donor age, methylprednisolone, mycophenolic acid, mycophenolic acid dose, hemoglobin, lymphocyte count, serum creatinine, iron, and 1α-OH-D3 use. The percentages of subjects with an adequate humoral response to SARS-CoV-2 were 43.2 and 65% for BNT162b2 and mRNA-1,273 vaccines, respectively (*p* = 0.152). Anti-N SARS-CoV-2 IgG antibodies in all patients were < 1.4 Index.

**Table 1:** Clinical characteristics of patients in kidney transplant recipients by anti-S SARS-CoV-2 antibody positivities.

Variable	Negative (Anti-S IgG < 50 AU/ml; *N* = 49, 52.1%)	Positive (Anti-S IgG ≥ 50 AU/ml; *N* = 45, 47.9%)	Total (*N* = 94)
Age, year	54.6 ± 11.7	52.8 ± 11.6	53.7 ± 11.7
Sex, male, *n* (%)	30 (61.2)	33 (73.3)	63 (67.0)
Body mass index, kg/m^2^	22.4 ± 3.8	23.4 ± 3.3	22.8 ± 3.6
Time post last transplantation, year	7.5 (2.6 to 14.9)	12.8 (9.0 to 17.7)	10.7 (4.4 to 16.9)
First transplant, *n* (%)	46 (93.9)	45 (100)	91 (96.8)
Donor type, living, *n* (%)	46 (93.9)	39 (86.7)	85 (90.4)
Donor age, year	59 (54 to 65)	51.5 (42.5 to 60)	57 (49 to 64)
Donor sex, male, *n* (%)	14 (30.4)	17 (40.5)	31 (35.2)
Primary kidney disease			
IgA nephrology	17 (34.7)	14 (31.1)	31 (33.0)
FSGS	4 (8.2)	4 (8.9)	8 (8.5)
Diabetes mellitus	5 (10.2)	2 (4.4)	7 (7.5)
Others	23 (46.9)	25 (55.6)	48 (51.0)
ABO incompatible, *n* (%)	9 (18.4)	5 (11.1)	14 (14.9)
HLA class I mismatch	2.0 ± 1.0	1.7 ± 0.9	1.9 ± 0.9
HLA class II mismatch	1.0 ± 0.5	0.9 ± 0.6	0.9 ± 0.5
Hypertension, *n* (%)	32 (65.3)	34 (75.6)	66 (70.2)
Diabetes mellitus, *n* (%)	14 (28.6)	11 (24.4)	25 (26.6)
Rituximab use, *n* (%)	15 (30.6)	12 (26.7)	27 (28.7)
Methylprednisolone use, *n* (%)	46 (93.9)	33 (73.3)	79 (84.0)
Tacrolimus use, *n* (%)	41 (83.7)	39 (86.7)	80 (85.1)
Mycophenolic acid use, *n* (%)	47 (95.9)	37 (82.2)	84 (89.4)
Mycophenolic acid dose, mg/day	1148.0 ± 450.5	838.9 ± 449.6	1000.0 ± 473.8
Methylprednisolone + Tacrolimus+ Mycophenolic acid, *n* (%)	37 (75.5)	29 (64.4)	66 (70.2)
Hemoglobin, g/dL	12.0 ± 2.2	13.7 ± 1.8	12.8 ± 2.1
Lymphocyte/μl	1,100 (800 to 1,500)	1,500 (1,100 to 2,300)	1,300 (1,000 to 1900)
Albumin, g/dL	4.1 ± 0.4	4.2 ± 0.3	4.2 ± 0.4
Serum creatinine, mg/dL	1.6 (1.1 to 2.0)	1.2 (1.0 to 1.5)	1.3 (1.0 to 1.9)
eGFR, ml/min/m^2^	37.0 ± 16.4	49.3 ± 14.8	42.9 ± 16.7
Iron drug use, *n* (%)	13 (26.5)	1 (2.2)	14 (14.9)
1α-OH-D3 drug use, *n* (%)	26 (53.1)	13 (28.9)	39 (41.5)
ACE-I or ARB use, *n* (%)	25 (51.0)	30 (66.7)	55 (58.5)
Rejection, *n* (%)	16 (32.7)	11 (24.4)	27 (28.7)
Times post second vaccine, day	140.9 ± 47.1	158.2 ± 42.3	149.2 ± 45.5
Antibody titers, AU/mL	4.9 (2.1 to 16.8)	194.9 (123.7 to 373.8)	35.3 (3.8 to 159.7)
Vaccine type, mRNA SARS-CoV-2 BNT162B2, *n* (%)	42 (85.7)	32 (72.7)	74 (79.6)

FSGS, Focal segmental glomerulosclerosis; ACE-I, angiotensin-converting enzyme inhibitor; ARB, angiotensin II receptor blocker. Values are *n* (%) or presented as means ± standard deviation or medians (interquartile range, 25th–75th percentiles).

In a multivariate logistic regression model, mycophenolic acid dose (OR = 0.998, 95% CI = 0.996 to 1.000), methylprednisolone (OR = 0.14, 95% CI = 0.02 to 0.85), high lymphocyte count (OR = 1.001, 95% CI = 1.000 to 1.002), and high hemoglobin level (OR = 1.67, 95% CI = 1.10 to 2.54) were associated with an appropriate humoral response (Table 2). Multivariate linear regression analysis showed that mycophenolic acid dose (*β* = −0.0004 95%, CI = −0.0009 to −0.00002), BMI (*β* = 0.06, 95% CI = 0.01 to 0.11), and time elapsed since the second dose (*β* = −0.005, 95% CI = −0.009 to 0.0002) were associated with an appropriate humoral response (Table 3).

**Table 2:** Factors associated with antibody response by univariate and multivariate logistic regression models.

Variable	Univariate odd ratio (95% CI)	*p* value	Multivariate odd ratio (95% CI)	*p* value
Age, year	0.99 (0.95 to 1.02)	0.45		
Male gender	1.74 (0.73 to 4.18)	0.21		
Body mass index, kg/m^2^	1.08 (0.96 to 1.21)	0.19		
Time post last transplantation, year	1.06 (1.01 to 1.12)	0.02	1.04 (0.93 to 1.15)	0.49
Treatment with rituximab	0.82 (0.34 to 2.02)	0.67		
Methylprednisolone	0.18 (0.05 to 0.69)	0.01	0.14 (0.02 to 0.85)	0.03
Mycophenolic acid dose, mg/day	0.998 (0.997 to 0.999)	0.003	0.998 (0.996 to 1.000)	0.04
Tacrolimus	1.27 (0.40 to 3.99)	0.68		
Hemoglobin, g/dL	1.55 (1.20 to 1.98)	0.001	1.67 (1.10 to 2.54)	0.02
Lymphocyte/μL	1.001 (1.0005 to 1.002)	0.001	1.001 (1.000 to 1.002)	0.01
eGFR, mL/min/1.73m^2^	1.05 (1.02 to 1.08)	0.001	1.02 (0.97 to 1.06)	0.47
Hypertension	1.64 (0.67 to 4.03)	0.28		
Diabetes mellitus	0.81 (0.32 to 2.03)	0.65		
Iron drug	0.06 (0.01 to 0.50)	0.009	0.31 (0.03 to 3.47)	0.34
1α-OH-D3 drug	0.36 (0.15 to 0.84)	0.02	0.94 (0.26 to 3.38)	0.93
Times post second vaccine, day	1.01 (1.00 to 1.02)	0.07	1.00 (0.98 to 1.01)	0.81

**Table 3:** Factors associated with anti-S SARS-CoV-2 antibody levels by univariate and multivariate liner regression models.

Variable	Univariate coefficient (95% CI)	*p* value	Multivariate coefficient (95% CI)	*p* value
Age, year	−0.01 (−0.03 to 0.004)	0.12		
Male gender	0.33 (−0.12 to 0.79)	0.15		
Body mass index, kg/m^2^	0.07 (0.01 to 0.13)	0.03	0.06 (0.01 to 0.11)	0.03
Time post last transplantation, year	0.02 (−0.01 to 0.04)	0.17		
Treatment with rituximab	0.08 (−0.41 to 0.56)	0.76		
Methylprednisolone	−0.65 (−1.23 to −0.08)	0.03	−0.49 (−0.99 to 0.13)	0.06
Mycophenolic acid dose, mg/day	−0.0006 (−0.001 to −0.0002)	0.01	−0.0004 (−0.0009 to −0.00002)	0.04
Tacrolimus	0.20 (−0.40 to 0.81)	0.50		
Hemoglobin, g/dL	0.19 (0.09 to 0.28)	<0.001	0.11 (−0.005 to 0.23)	0.06
Lymphocyte/μl	0.0006 (0.0003 to 0.001)	<0.001	0.0003 (−0.000006 to 0.0006)	0.06
eGFR, mL/min/1.73m^2^	0.02 (0.01 to 0.03)	<0.001	0.14 (−0.001 to 0.03)	0.07
Hypertension	0.25 (−0.23 to 0.72)	0.30		
Diabetes mellitus	0.07 (−0.42 to 0.56)	0.77		
Iron drug	−0.90 (−1.50 to −0.30)	0.004	−0.16 (−0.73 to 0.41)	0.58
1α-OH-D3 drug	−0.44 (−0.87 to −0.01)	0.046	−0.20 (−0.60 to 0.20)	0.33
Times post second vaccine, day	0.001 (−0.004 to 0.006)	0.77	−0.005 (−0.009 to −0.0002)	0.04

## Discussion

The total number of kidney transplants in Japan is 36,373, and the number of living patients is approximately 22,000 according to the 2020 Factbook of the Japanese Society of Transplantation (Fact Book, 2020). For KTRs, the COVID-19 mortality rate is reportedly 22 to 32%, higher than in the general population (Cravedi et al., 2020; Coll et al., 2021; De Meester et al., 2021). In the general population, BNT162b2 and mRNA-1,273 vaccines have an efficacy rate of 92 to 94.1% (Baden et al., 2021; Dagan et al., 2021). However, in KTRs, the rate of an appropriate humoral response is only 30.0 to 52.4% at 1 month after the second dose, and the antibody titer is low (Benotmane et al., 2021b; Boyarsky et al., 2021b; Cucchiari et al., 2021; Grupper et al., 2021; Rozen-Zvi et al., 2021; Russo et al., 2022). On this basis, the United States, Canada, Germany, and France gave priority to KTRs for a third dose. In Germany and Canada, an appropriate humoral response was observed in 61.2 and 60% of KTRs at 1 month after the third dose, respectively (Bertrand et al., 2021; Hall et al., 2021). In France, an appropriate humoral response was observed in 44 to 49% of KTRs (who did not respond to a second dose) at 1 month after the third dose (Benotmane et al., 2021a; Kamar et al., 2021). In this study, the long-term appropriate humoral response rate was 47.8% and the anti-S SARS-CoV-2 IgG antibody titers was very low at 35.3 (IQR = 3.8 to 159.7) AU/mL at 5 months (149.2 ± 45.5 days) after the second dose. Similarly, the appropriate response rate was 42% in Israel (Ben-Dov et al., 2022). Therefore, third doses should be administered to Japanese KTRs. In Japan, as of 1 March, 2022, the rate of third-dose vaccination is 20.4%, which does not differ according to pre-existing condition. In France, administration of a fourth dose elicited an appropriate humoral response in KTRs (Benotmane et al., 2022). The two vaccines may be less effective against omicron; however, additional doses elicit a response (Garcia-Beltran et al., 2022). Indeed, the CDC recommends a fourth dose for KTRs (Centre for Disease Control and Prevention, 2021).

The factors associated with an appropriate humoral response in a multivariate logistic model were a low mycophenolic acid dose, non-use of methylprednisolone, a high hemoglobin level, and a high lymphocyte count. The factors identified in a multivariate linear regression model were mycophenolic acid dose, BMI, and time elapsed since the second dose. Therefore, only a low mycophenolic acid dose was associated with an appropriate humoral response in KTRs. A high lymphocyte count (Ben-Dov et al., 2022) and fewer types of immunosuppressants (Grupper et al., 2021; Rozen-Zvi et al., 2021), are, as would be expected, reportedly associated with the lack of an appropriate humoral immune response. Data on the associations of hemoglobin and BMI with a humoral response in KTRs are sparse. Advanced age, decreased renal function, steroid pulse therapy, diabetes, history of cancer, serum ferritin level, and serum 25(OH)VitD level have also been reported to be associated with an inappropriate humoral immune response (Grupper et al., 2021; Rozen-Zvi et al., 2021; Ben-Dov et al., 2022). Although these factors were not associated with an appropriate humoral response in this study, analysis of a larger number of cases might have yielded associations.

The antibody positivity rate was not significantly different between the two vaccines. The rate of an appropriate humoral response rate for the BNT162b2 and mRNA-1,273 vaccines in KTRs was 46% (Boyarsky et al., 2021b) and 48% (Benotmane et al., 2021b) at 1 month after the second dose, 61.2% (Bertrand et al., 2021) and 60% (Hall et al., 2021) at 1 month after the third dose, and 44% (Kamar et al., 2021) and 49% (Benotmane et al., 2021a) in those who did not show an appropriate response to the second dose, respectively. These data were obtained from subjects of different races and nationalities. By contrast, the mRNA-1,273 vaccine reportedly has a higher antibody positivity rate than the BNT162b2 vaccine (Wijtvliet et al., 2021; Haller et al., 2022; Marinaki et al., 2022). Thus, additional studies are needed to evaluate the rates of an appropriate humoral response between the two vaccines.

The limitations of this study include its single-center design and small number of cases. In addition, we focused on antibody titers and did not evaluate cellular immunity or the link between antibody titer and prevention of infection or severe disease. Heterogeneity in antibody titer measurement techniques frankly limits quantitative comparison of results from different studies. Further study of the effects of third and fourth doses on antibody titers and the prevention of infection and severe disease is warranted.

In conclusion, less than half of KTRs had an appropriate humoral response and the antibody titer was low at 149.2 ± 45.5 days after the second dose of the mRNA SARS-Cov-2 vaccine. As in other countries, 5 months is too long to wait for a 3rd dose after 2nd dose of SARS-CoV-2 vaccine in KTRs. A third or fourth dose of the mRNA SARS-Cov-2 vaccine may be necessary in KTRs, who are susceptible to severe disease.

## Data Availability Statement

The raw data supporting the conclusions of this article will be made available by the authors, without undue reservation.

## Ethics Statement

The studies involving human participants were reviewed and approved by The Ethics Committee of the School of Medicine of Jikei University [approval number 33–314(10934)] approved the study protocol. The patients/participants provided their written informed consent to participate in this study.

## Author Contributions

YO, MK, IY, and HY participated in the clinical practice, designed the study protocol, and drafted the manuscript. TY, HK, YN, AK, NM, YT, TH, and IO participated in the patient’s care and revised the manuscript. FU, JM, HY, and TK performed the kidney transplantation. TY is the divisional directors and supervised each author. All authors contributed to the preparation of the manuscript and approved the final version.

## Conflict of Interest

The authors declare that the research was conducted in the absence of any commercial or financial relationships that could be construed as a potential conflict of interest.

## Publisher’s Note

All claims expressed in this article are solely those of the authors and do not necessarily represent those of their affiliated organizations, or those of the publisher, the editors and the reviewers. Any product that may be evaluated in this article, or claim that may be made by its manufacturer, is not guaranteed or endorsed by the publisher.
